# Role of CD34 in inflammatory bowel disease

**DOI:** 10.3389/fphys.2023.1144980

**Published:** 2023-03-27

**Authors:** Zhiyuan Li, Shuyan Dong, Shichen Huang, Yuhan Sun, Yingzhi Sun, Beibei Zhao, Qiulan Qi, Lei Xiong, Feng Hong, Yuxin Jiang

**Affiliations:** ^1^ Jiaxing Key Laboratory of Virus-Related Infectious Diseases, The Affiliated Hospital of Jiaxing University, Jiaxing University College of Medicine, Jiaxing, Zhejiang, China; ^2^ School of Pharmacy, Wannan Medical College, Wuhu, Anhui, China; ^3^ Department of Biochemistry and Molecular Biology, Wannan Medical College, Wuhu, Anhui, China; ^4^ The Brain Cognition and Brain Disease Institute, Shenzhen Institute of Advanced Technology, Chinese Academy of Sciences, Shenzhen, Guangdong, China

**Keywords:** CD34, inflammatory bowel disease, immune cells, adhesion molecules, selectin, integrin

## Abstract

Inflammatory bowel disease (IBD) is caused by a variety of pathogenic factors, including chronic recurrent inflammation of the ileum, rectum, and colon. Immune cells and adhesion molecules play an important role in the course of the disease, which is actually an autoimmune disease. During IBD, CD34 is involved in mediating the migration of a variety of immune cells (neutrophils, eosinophils, and mast cells) to the inflammatory site, and its interaction with various adhesion molecules is involved in the occurrence and development of IBD. Although the function of CD34 as a partial cell marker is well known, little is known on its role in IBD. Therefore, this article describes the structure and biological function of CD34, as well as on its potential mechanism in the development of IBD.

## 1 Introduction

The incidence of inflammatory bowel disease (IBD) has increased worldwide in recent years due to the change in eating habits and living conditions of people, severely affecting the quality of life (Ramos and Papadakis, 2019; van Gennep et al., 2021). IBD is a non-specific chronic intestinal inflammatory disease that includes ulcerative colitis and Crohn’s disease (Jain and Venkataraman, 2021). Both of them are immune-mediated diseases of the digestive system, characterized by inflammation, diarrhea, abdominal pain, rectal bleeding, and weight loss (Seyedian et al., 2019). Ulcerative colitis is more commonly affecting the rectum and colon mucosa, leading to inflammation and ulcers (Conrad et al., 2014). Crohn’s disease affects the entire digestive system from the mouth to the anus, often leading to inflammation of the entire intestinal layer (transmural and skip lesions), even further affecting the physiological functions of other organs through fistulas (M’Koma, 2022; Nosti et al., 2013). However, the pathogenesis and etiology of IBD are still unclear. The imbalance of the intestinal homeostasis is an important factor leading to IBD due to the important role of the intestinal microbiota in the maintenance of this homeostasis. The destruction or the change of the intestinal microbiota microenvironment often leads to mucosal immune disorders (Xavier and Podolsky, 2007; Torres and Rios, 2008), which cause an immune response that often represents the beginning of an inflammation. Various immune cells (Margraf and Perretti, 2022; Siel et al., 2022) and adhesion molecules (Reglero-Real et al., 2016) are known to be involved in the regulatory role of IBD, but their significance in the pathogenesis of IBD is still unclear (Torres and Rios, 2008).

Inflammation is a defensive reaction of the living tissue possessing a vascular system against harmful factors. The local reaction is characterized by redness, swelling, heat, and pain, and the systemic reaction is leukocytosis. During the inflammatory response, leukotrienes (LTs) produced by lipoxygenase catalysis act as inflammatory mediators by causing endothelial cell contraction and increasing vascular permeability. The study of Singh et al. (2004) revealed that the expression of this factor is significantly increased in IBD rats, and it promotes the release of adhesion molecules such as selectin and integrin from vascular endothelial cells, which participate in IBD (Cuzzocrea et al., 2005). For example, a large number of cell adhesion molecules is expressed on mucosal microvascular endothelial cells when intestinal inflammation occurs, so that white blood cells are further recruited into the inflammatory site, aggravating the inflammatory response (Liu et al., 2021b). In addition, leukotriene B4 is a potent immune cell chemotactic factor that causes the accumulation of immune cells at sites of inflammation (Castellani et al., 2009). These pieces of evidence suggest that the expression of inflammatory factors and the above-mentioned adhesion molecules mediated by inflammatory factors are involved in the occurrence and development of inflammation.

CD34 is a type I transmembrane phosphorylated glycoprotein with a relative molecular mass of approximately 115 KDa. It is a member of the cluster of differentiation and, as such, it is expressed in mucosal dendritic cells, mast cells, eosinophils, and other immune cells (Aulakh et al., 2021a). CD34 has a stable molecular structure and is often used as a cell marker for hematopoietic stem cell sorting, as well as islet endocrine cell marker (Huang et al., 2022). It has also been extensively studied in cell adhesion, inflammatory cell chemotaxis, cell proliferation and differentiation, and enhancement of the inflammatory response (Nielsen and McNagny, 2008; Grassl et al., 2010). Abnormally high CD34 expression is present in the intestine of IBD patients, (Tsiolakidou et al., 2008), and this increased expression has a certain role in the immune regulation of IBD. Indeed, immune cells such as neutrophils (Aulakh, 2020), eosinophils (Maltby et al., 2010), and mast cells (Drew et al., 2002) are recruited to the intestinal inflammation site through CD34. CD34 interacts with selectin (Lo et al., 2017) and integrin (Ohnishi et al., 2013) and exerts a synergistic effect with inflammatory cytokines, chemokines (Kitajima et al., 1999; Maltby et al., 2010) and other immune substances, thus participating to intestinal inflammation and promoting the progression of IBD (Milia et al., 2013; Ohnishi et al., 2013). The role of immune cells through CD34 is essentially the result of the interaction between cell adhesion molecules and CD34. In addition, CD34^+^ interstitial cells in the intestine are involved in the repair of the intestinal tissue damage (Wu et al., 2021). These loads of evidence suggest that CD34 plays an important role in the development of IBD.

Although the structure and biological functions of CD34 are well known, its role in the intestinal inflammatory response remains unclear (Hughes et al., 2020). Therefore, this article describes the biological function of CD34 involved in the migration of some immune cells and interaction with adhesion molecules, as well as the potential mechanism associated with its role in the inflammatory response during IBD.

## 2 Structure and localization of CD34

CD34 is located in the cell surface of hematopoietic cells, stromal cells, epithelial cells, and endothelial cells (Sidney et al., 2014), belongs to the class I transmembrane protein molecule, and possesses a structure that includes an extracellular region, transmembrane region, and cytoplasmic region (Hughes et al., 2020). The extracellular region is composed of 278 amino acid residues, with a certain degree of conservation and it is the main region interacting with other molecules. The extracellular domain of CD34 is rich in chain sugars that maintain its structural stability, protecting it from the hydrolysis by protease and providing specific recognition sites. The terminal 145 amino acid residues contain a high proportion of serine and threonine (up to 35%), which form a cluster of sialylation sites that are essential for its adhesion molecule function. In addition, the highly conserved amino acid sequence of the CD34 extracellular structure suggests that its biological function can be traced back to very ancient times. The transmembrane domain of CD34 is represented by an α-helix structure composed of 22 hydrophobic amino acid residues characterized by a type I transmembrane protein structure. The cytoplasmic region is composed of 73 hydrophobic amino acid residues recognizable by the C-terminal SH3 domain of the regulatory Crk-like [Crk(Cysteine-rich receptor-like kinase protein)-like, CrkL] protein (CrkL-SH3C) and this cytoplasmic region plays a role in inducing cell aggregation. The extracellular region of CD34 determines its function of mediates cell recognition and adhesion, and the cytoplasmic region determines its ability to mediate migration and aggregation, providing the basic conditions for its participation in the inflammatory response (Sidney et al., 2014). In addition, CD34 expressed in many tissues and organs of human such as spleen, lung, and pancreatic islets and gastrointestinal tract (Huang et al., 2022). The expression of CD34 in the gastrointestinal tract is higher compared with its expression in other organs, suggesting a major involvement of CD34 in the gastrointestinal tract functions. During IBD, CD34 works as an adhesion molecule or acts as an adhesion molecule ligand (Milia et al., 2013; Ohnishi et al., 2013) to mediate the location of various immune cells (mast cells, neutrophils, and eosinophils) at the inflammatory site (Aulakh et al., 2021a). These aspects prompted us to further explore the function of CD34 in IBD.

## 3 CD34 and IBD

Although CD34 is widely used as a marker of hematopoietic stem/progenitor cells in clinical and scientific research, its specific biological function has not been fully confirmed (Sidney et al., 2014). CD34 has been repeatedly mentioned in studies on intestinal inflammation and intestinal function. Indeed, CD34 expression in the intestinal mucosa of IBD patients is significantly higher than that in healthy subjects (Tsiolakidou et al., 2008); inflammation is relatively mild in CD34 knockout mice belonging to the dextran sulfate-induced ulcerative colitis mouse model (Maltby et al., 2010); another report showed that the treatment with curcumin reduces the levels of CD34 immunoreactive capillaries and inflammatory cells in the intestinal inflammatory site compared with its levels in the control group (Hamed et al., 2022). Since CD34 is also involved in the recruitment of various immune cells in the inflammatory site during IBD (Grassl et al., 2010; Aulakh et al., 2021b), these results demonstrated that CD34 played a migration role in the process of immune cells reaching the site of inflammatory bowel disease.

Different types of IBD are characterized by different CD34 expressions. Indeed, its expression in ulcerative colitis is higher than that in Crohn’s disease. Thus, this difference has been used as a criterion to distinguish these two diseases and applied to clinical diagnosis in some cases (Zurawski et al., 2007). During intestinal inflammation, CD34^+^ cells are significantly increased in the submucosa, endothelium, and lamina propria of the intestinal tissue (Grassl et al., 2010). In addition, the telocytes (TCs) presented in the intestinal wall of the human colon are involved in ulcerative colitis, since colonic motor dysfunction occurs when TCs in the intestinal wall of patients with advanced fibrosis ulcerative colitis decrease or even disappear (Manetti et al., 2015). CD34 has been used as a marker of TCs (Zurzu et al., 2021); the number of CD34^+^ TCs in the intestine of rats with ulcerative colitis is significantly decreased, and it gradually increases after drug treatment and the consequent improvement of the symptoms (Arafat et al., 2021). Other studies showed that CD34 is expressed in fibroblasts at the site of the intestinal injury, promoting the inflammatory response and repairing the damaged tissues (Sidney et al., 2014; AbuSamra et al., 2017). Thus, CD34 molecule and CD34^+^ cells play a role in regulating intestinal inflammation, but their specific mechanism remains unclear.

CD34 is currently widely studied in a variety of gastrointestinal diseases. It is highly expressed in gastrointestinal stromal tumor cells, and its combined detection with CD117 allows the distinction of gastrointestinal stromal tumors from smooth muscle tumors and schwannoma (Miettinen and Lasota, 2006a; Miettinen and Lasota, 2006b). In addition, gastrointestinal stromal tumors may originate from the Cajal CD34^+^ stromal cell subset (Robinson et al., 2000). CD34 is abnormally expressed in the tumor microenvironment of colorectal cancer patients; when the cytoskeleton class VI intermediate filament protein nestin and CD34 are both overexpressed, patients survival rate is significantly improved, suggesting that nestin and CD34^+^ expression in the interstitial cells in colorectal cancer possess a potential protective role, which may be related to the immune function of the body (Tampakis et al., 2021). All these pieces of evidence demonstrated that the pathogenesis of IBD and its related diseases are more or less associated with CD34.

## 4 CD34 and immune cells

### 4.1 CD34 and granulocytes

Granulocytes are a class of leukocytes that contain special staining granules in their cytoplasm. Based on the staining granules, granulocytes are classified as neutrophils, eosinophils and basophils. Of these, neutrophils and eosinophils are most closely related to the occurrence and development of inflammatory bowel disease. Neutrophils and eosinophils are most closely related to the development of IBD (Siel et al., 2022). Neutrophils are components of the innate immune system and their recruitment and aggregation during IBD occur in the intestinal mucosa (Sadik et al., 2011; Chen et al., 2021). They have both positive and negative effects on IBD; on the one hand, they promote the repair of mucosal inflammatory injury by removing harmful pathogens (Sumagin et al., 2016). On the other hand, they are involved in the occurrence and aggravation of inflammation in the intestinal mucosal by participating in the excessive immune response (Wandall, 1985; de Bont et al., 2020). Neutrophil recruitment and infiltration in the intestinal mucosa of patients with IBD is the result of the combination of cytokines and chemokines presence (Chen et al., 2021). Notably, the literature suggests that CD34 recruits neutrophils to the sites of inflammation (Grassl et al., 2010; Lo et al., 2017). Indeed, the recruitment rate of neutrophils in CD34 knockout mice slowed down, resulting in prolonged recruitment time of neutrophils to inflammatory sites and aggravation of inflammation (Aulakh, 2020). Similar results are reported by Aulakh et al. in their study of endotoxin-induced inflammation (Aulakh et al., 2021b). These studies suggest that CD34 may also affect the inflammatory response by mediating the recruitment and infiltration of neutrophils in the inflammatory site during IBD.

Eosinophils are also involved in the pathogenesis of IBD; hence, they are worthy of attention (Carvalho et al., 2003). Eosinophils are a class of cells that are differentiated from CD34^+^ bone marrow progenitor cells (Uchida et al., 2022), and have a role in the immune response. During IBD, eosinophils mainly accumulate in the intestinal mucosa of mice. Eosinophils, like neutrophils, exert two opposite effects. On the one hand, they protect the colon during acute colitis in mice by producing anti-inflammatory substances (Masterson et al., 2015). On the other hand, eosinophils accumulate in the intestinal mucosa, and the synthesis and release of inflammatory mediators by themselves cause intestinal tissue damage (Wędrychowicz et al., 2014; Mehta and Furuta, 2015). Eosinophils express CD34 in colon inflammation (Maltby et al., 2010) due to the extracellular CD34 domain richness in serine, threonine, and proline residues, and CD34 domain can be widely subjected to O-glycosylation and sialylation (Nielsen and McNagny, 2008), meaning that it participates in the intestinal inflammation through this special molecular structure. CD34 enhances the migration ability of eosinophils, since those lacking CD34 have impaired migration, and the symptoms of ulcerative colitis are consequently alleviated (Suzuki et al., 1996; Blanchet et al., 2007). This suggests that CD34 influences the effect of eosinophils on the pathogenesis of IBD, although the specific molecular mechanism remains unclear.

### 4.2 CD34 and mast cells

Mast cells are multifunctional immune cells working in both innate and adaptive immunity and developed from CD34^+^ bone marrow progenitor cells (Weinstock et al., 2021). Mast cell number in the intestine of inflamed IDB mice is significantly increased compared to their number in control mice (Kurashima et al., 2012). Tryptase secreted by mast cells during IBD causes intestinal fibrosis and aggravates the degree of IBD (Liu et al., 2021a); inflammatory mediators released by mast cells also affect important physiological functions such as intestinal permeability, motility, secretion, and mucosal immune response (Hamilton et al., 2014). According to Dr. Boeckxstaens’s research, mast cells accumulate on the surface of the intestinal mucosa and mediate inflammatory responses through ATP and P2X7 purinergic receptors. During this process, they can promote the infiltration of neutrophils at the site of inflammation, further exacerbating the inflammatory response (Boeckxstaens, 2015). Activated mast cells are blocked when IBD mice are treated with the immunoglobulin light chain antagonist F991, and the inflammatory symptoms are improved (Beunk et al., 2013). The above evidence suggests that mast cells are involved in the occurrence and development of IBD and the pathological changes of the inflammatory sites. Mast cells exert different effects during IBD related to their migration, adhesion, or mediation of other immune cells to the site of inflammation (Hiromatsu and Toda, 2003; Méndez-Enríquez and Hallgren, 2019). As we mentioned above, CD34 is involved in cell adhesion, inflammatory cell migration, and chemotaxis (Nielsen and McNagny, 2008; Grassl et al., 2010); this glycoprotein is mentioned many times in studies on the migration and adhesion of mast cells in the site of inflammation (Drew et al., 2002; Nielsen and McNagny, 2009), and CD34 expression in the intestinal mucosal mast cells is significantly higher than that in other sources of mast cells. These results suggest that CD34 in IBD affects the migration and adhesion of mast cells, thereby promoting their infiltration in the inflammatory site and the regulation of the immune response.

CD34 was originally studied as a marker, and the initial reports indeed revealed the link between CD34 and mast cells as a marker of the mast cell surface (Drew et al., 2002). However, further studies discovered that CD34 promotes mast cell migration and blocks mast cell adhesion in IBD (Nielsen and McNagny, 2009). The migration of mast cells is inhibited when CD34 not expressed, suggesting that CD34 promotes the migration of mast cells to the inflammatory sites (Drew et al., 2002; Nielsen and McNagny, 2009). In addition, CD34 prevents the aggregation between mast cells, as revealed by a study of mast cell adhesion. The adhesion ability between mast cells is enhanced and the aggregation is increased when CD34 not expressed, (Drew et al., 2005), which also explains the phenomenon explained above on the inhibition of mast cell migration. The homology aggregation of CD34-deficient mast cells is more significant when the gene encoding sialic acid is knocked out (Drew et al., 2005), suggesting that CD34 plays a role in blocking mast cell adhesion and aggregation. The function depends on the sialylation of its extracellular domain, which further links its cell function to its molecular structure. In addition, studies have found that interstitial cell of Cajal (ICC), which regulate the function of intestinal smooth muscle, are associated with more than a dozen gastrointestinal diseases, and ICC in rat intestinal tissue show CD34 immunoreactivity. Furthermore, ICC interacts with mast cells (Yu et al., 2012; Tampakis et al., 2021) in patients with Crohn’s disease; all subtypes of ICC have intermembrane contact with mast cells, while ICC has little contact with other types of immune cell membranes. The interaction between ICC and mast cells causes structural damage (Wang et al., 2007). However, at present, the molecular mechanism of the connection between mast cells and ICC or CD34^+^ ICC is still under study.

## 5 CD34 and adhesion molecules

The previous immune cell focuses on the relationship between CD34 and various immune cells and its potential impact on the IBD process. However, in addition to the above relationship, IBD is also characterized by the interaction between various biomolecules, and among them, the most important are the adhesion molecules. Indeed, the involvement of various cells in the inflammatory response is essentially the result of the signal transduction regulated by various biomolecules through various pathways. Therefore, the role and relationship between several adhesion molecules involved in the inflammatory response and CD34 is discussed to explain the molecular mechanism used by CD34 to mediate the migration of immune cells to the inflammatory sites in IBD.

### 5.1 CD34 and selectin

Selectins are a class of heterophilic, Ca^2+^-dependent biomolecules with a highly conserved lectin domain in their extracellular region. They specifically recognize glycosyl ligands at the end of oligosaccharide chains on other cell surfaces to exert their function. They belong to the family of adhesion molecules mainly involved in the recognition and adhesion between leukocytes and vascular endothelial cells (Zak et al., 2000). The selectin subfamily includes three members: platelet (P)-selectin, endothelial (E)-selectin, and leukocyte (L)-selectin with an N-terminal lectin-like domain, epidermal growth factor-like domain, variable number of common repeats, a single transmembrane domain and a short cytoplasmic tail (Ley, 2003; Rosen, 2004). White blood cells recognize and adhere to vascular endothelial cells, migrating to the inflammatory site. The molecular basis of this mechanism is the expression of adhesion molecule regulated by cytokines during inflammation and the consequent interaction between leukocytes and adhesion molecules on the surface of vascular endothelial cells. The physiological functions of leukocytes such as their rolling and adhesion on endothelial cells are mediated by the binding of selectins on leukocytes to the corresponding glycosylated ligands on endothelial cells (Lawrence and Springer, 1991; Kaplanski et al., 1993). In other words, the interaction between the selectin molecule and its glycosylation ligand promotes the rolling, adhesion, and other functions of leukocytes on the vascular endothelium, consequently starting a series of reactions, ultimately causing the aggregation of leukocytes in the inflammatory response area. Although all the three selectins bind to their corresponding glycosylation ligands through the N-terminal lectin-like domain (Patel et al., 1995), they differ in the ligands they prefer. L-selectin, which is widely studied in the inflammatory response, mainly binds to glycoprotein ligands including CD34 (Lanza et al., 2001; Smith and Bertozzi, 2021). The extracellular domain of CD34 contains multiple O-linked glycans, making it highly specific for L-selectin (Rosen, 2004). During inflammation, L-selectin on the plasma membrane of the lymphocytes recognizes the specific carbohydrate chain of CD34 and mediates the rolling of immune cells on endothelial cells (Nielsen and McNagny, 2008). CD34 only acts as a ligand of L-selectin; the binding of L-selectin to CD34 occurs when it is glycosylated to sulfated carbohydrate 6-sulfanilic, while the binding of CD34 is β-sialic acid-specific and Ca^2+^ dependent. This binding mediates the rolling of immune cells (Baumhueter et al., 1994; Krause et al., 1996; Hernandez Mir et al., 2009), suggesting that CD34 molecules are post-translationally modified in a specific environment to initiate the reaction.

**FIGURE 1:**
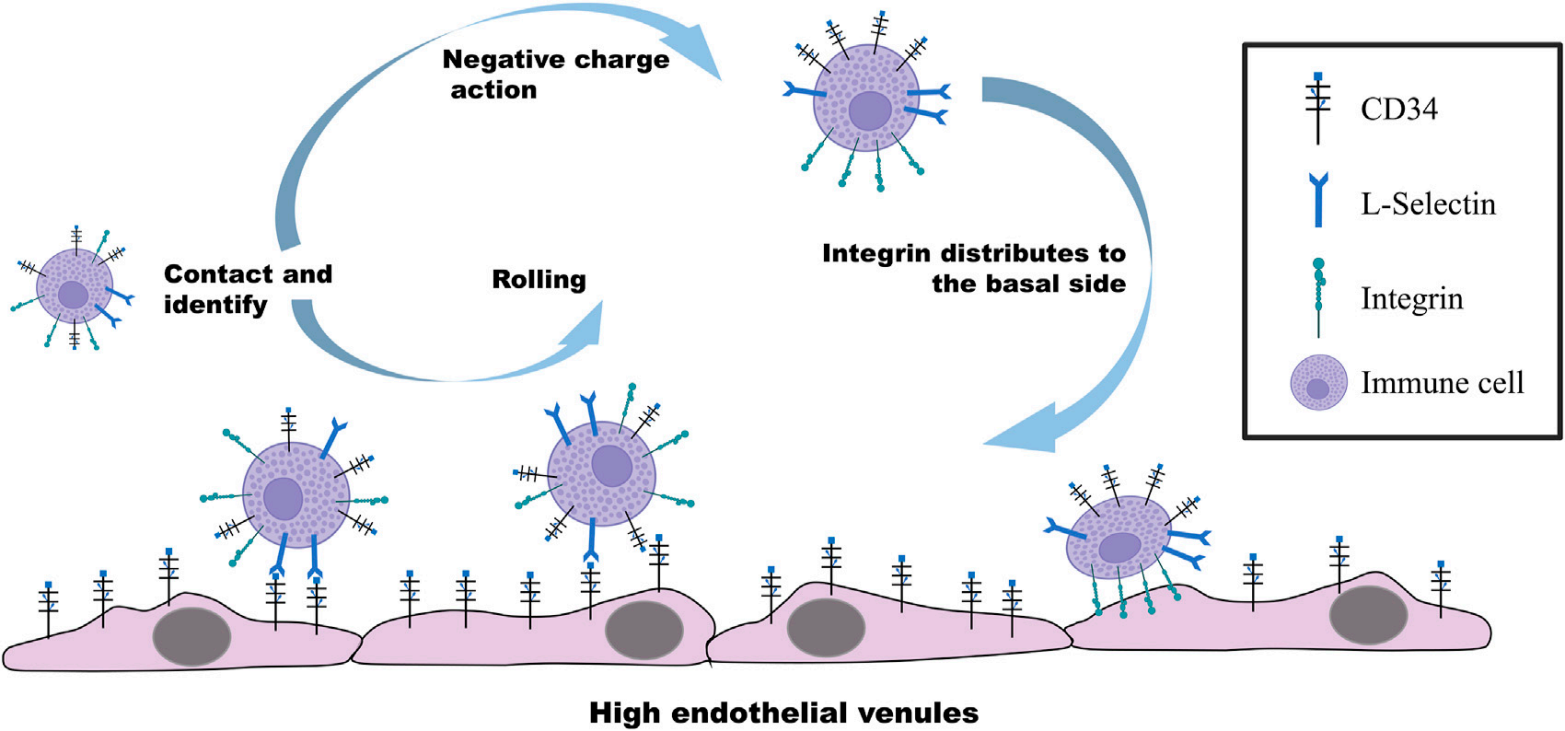
Molecular mechanism regulating the rolling and adhesion of immune cells on vascular endothelial cells. CD34-specific glycosylation on the surface of endothelial cells under the stimulation of inflammatory factors recognizes and binds to L-selectin on immune cells. Immune cells driven by the blood flow roll on vascular endothelial cells with the binding force of CD34 and L-selectin. When immune cells are in contact with endothelial cells, CD34 on the surface of the immune cells distributes integrin on their basal region through the negative charge carried by the extracellular region, further improving the contact with endothelial cells to exert the adhesion.

In recent years, the interaction between CD34 and L-selectin has been repeatedly reported in IBD studies. CD34, with its unique glycosylated structure as the ligand of L-selectin on lymphocytes, promotes the migration, adhesion, and aggregation of lymphocytes to the intestinal inflammatory sites during IBD (Maltby et al., 2010), participating in the initial stage of the inflammatory response, as shown in Figure 1. The level of soluble L-selectin increases with the aggravation of ulcerative colitis symptoms, while the level of L-selectin in Crohn’s disease is slightly higher but not significant than ulcerative colitis (Seidelin et al., 1998). Interestingly, the expression of CD34 in ulcerative colitis and Crohn’s disease is also correlated (Zurawski et al., 2007). The pieces of evidence suggest that the increase in CD34 expression during inflammatory diseases further leads to the increase in L-selectin level in the inflammatory site, that is, the inflammatory site affects the level of immune cells such as lymphocytes by regulating the content of CD34.

The extracellular domain of CD34 is an ubiquitin ligand after glycosylation modification, and it works as a ligand to bind the three selectins (Aulakh, 2020). CD34 has attracted more attention as a ligand for L-selectin due to the difference in affinity caused by different molecular structures. However, the interaction between CD34 and E-selectin and P-selectin during IBD has also been reported as of great significance in the development of this disease (AbuSamra et al., 2017). Similar to the bind of L-selectin with CD34, the CD34 molecule interacts with E-selectin and P-selectin by the corresponding selectin site on the surface of lymphocytes through the side chain structure, allowing the migration and aggregation of lymphocytes to the inflammatory site and initiating the inflammatory response (Epstein et al., 2006). Overall, the interaction between CD34 and selectins is of great significance in the occurrence and development of IBD, revealing its role in inflammatory diseases. However, the identification of specific ligands/receptors between CD34 and selectins should be investigated more deeply, and since the binding kinetics, affinity, and biological pathways involved in the process of IBD need to be further explored.

### 5.2 CD34 and integrins

Integrins are heterophilic cell adhesion molecules that are ubiquitous on the surface of vertebrate cells and their regulation depends on Ca^2+^ or Mg^2+^. Integrins are composed of two different subunits, α (120–185 kD) and β (90–110 kD) joined by non-covalent binding. To date, 18 α subunits and 9 β subunits have been found have been identified, which are able to generate 20 different integrins. Different subunits correspond to different functions of integrins (Dedhar, 1999; Takada et al., 2007). Specific ligands mediate the recognition and adhesion among cells and between cells and extracellular matrix by the binding to the integrin subunits (Campbell and Humphries, 2011). According to the specificity of the integrin recognition ligand or the composition of its subunits, integrins are divided into different subtypes with different functions (Takada et al., 2007; Barczyk et al., 2010). Integrins are important targets in the treatment of IBD. For example, the inhibition of α4β7 integrin by the lymphocyte α4β7 inhibitors blocks leukocyte transport during inflammation and improves IBD symptoms (Misselwitz et al., 2020); the treatment of Crohn’s disease with monoclonal antibodies (efalizumab and natalizumab) prepared with the αL and α4 subunits of the integrin as antigens resulted in a significant improvement of the symptoms (James et al., 2011); In addition, novel antibodies and small molecule drugs targeting β7 integrins (α4β7 and αEβ7) are under development and have potential for clinical treatment of inflammatory bowel disease (Ley et al., 2016). However, the specific molecular mechanism regulating the involvement of integrin in the development of IBD remains to be further clarified.

CD34 belongs to salivary mucin. The fine structure of the glycosylation moiety contained in its N-terminal region determines its interaction with various ligands, and extracellular mucin domain of CD34 has a highly conserved O-glycan chain. The structure of CD34 composed of a large number of hydroxyl and carboxyl groups makes the molecule hydrophilic with a large number of negative charges (Lanza et al., 2001). When non-adherent and non-polar cells cultured in suspension express both CD34 and integrin, the negatively charged CD34 is rapidly distributed in the apical domain once the cells establish the contact with the basement or basement membrane, and the integrin is moved to the lateral region of the basement to further enhance the adhesion. Indeed, during this process, CD34 is not functioning as an adhesion molecule but plays a role in moving integrins from the apical region of the cell to the basal lateral region, promoting their distribution and aggregation in the new location and exerting adhesion function in the basal lateral region (Ohnishi et al., 2013; Hughes et al., 2020). This mechanistic behavior shown in Figure 1 reasonably explains the ability of adhesion molecules to enhance cell-matrix binding and the involvement of CD34 in the cell adhesion mediated by integrins (Ohnishi et al., 2013). In recent years, many studies reported the function of CD34 as a promoter of the adhesion function of integrins in the basal region of intestinal cells and develop IDB. For example, α4β7 integrins present in most intestinal mucosal lymphocytes regulate the aggregation of lymphocytes in the intestine (Lobatón et al., 2014; Ley et al., 2016), and β2 subunit-based integrins are mainly present on the surface of various leukocytes, promoting immune cells into the intestinal mucosa (Foroutan et al., 2019). In these inflammatory processes, CD34 promotes the adhesion of basal integrins to the extracellular matrix by moving them from the apical region of cells (Hughes et al., 2020). The specific effect of CD34 on integrin may be due to the large amount of negative charge present in its extracellular structure, although no direct evidence is available on this aspect. In addition, although many types and subtypes of integrins exist, no systematic reports are available on the interaction between CD34 and various integrin subtypes and their potential application in IBD treatment.

### 5.3 CD34 and other adhesion molecules

Adhesion molecules are molecules of different types that mediate the contact and binding among cells or between cells and the extracellular matrix. In addition to selectins and integrins discussed above, there are the immunoglobulin superfamily, cadherin family, mucin-like vascular addressin family, and some unclassified adhesion molecules (Meirelles Lda and Nardi, 2009). Adhesion molecules are inflammatory mediator that are involved not only in the inflammatory response, but also in cell adhesion, innate immune response, and acquired immune response (Reglero-Real et al., 2016). According to the above evidence, the adhesion molecules of the selectin and integrin families are involved in IBD, but other adhesion molecules of other families are also worthy of attention because of their potential involvement in IBD. Indeed, vascular cell adhesion molecule-1 (VCAM-1), a member of the immunoglobulin superfamily (IgSF) localized on endothelial cells at the site of inflammation, participates in the inflammatory response after binding to integrin α4β1 on the surface of lymphocytes (Takada et al., 2007). In addition, the IgSF member triggering receptor expressed on myeloid cell-2 (TREM-2) enhances the inflammatory response and increases the pathological damage on the inflammatory site during intestinal inflammation. The examination of the inflammatory site in IBD patients revealed that the expression of TREM-2 in the diseased intestinal mucosa is significantly increased. The susceptibility to colitis is reduced When the *TREM-2* gene is deleted and the symptoms are less intense under the same conditions (Correale et al., 2013; Genua et al., 2014). Not only the immunoglobulin superfamily is associated with the occurrence of inflammation in ulcerative colitis, but also the cadherin family and the calmodulin-binding protein. For example, the high expression of protocadherin during the regeneration of the intestinal epithelial cells allows the orderly growth of cells and the repair of the inflammatory damage in the tissues (Fogt et al., 2008). In addition, vascular addressin located on the surface of the intestinal vascular endothelial cells is another IBD-associated adhesion molecule that participates in the intestinal inflammatory immune responses by mediating lymphocyte homing (Butcher and Picker, 1996).

Inflammation is the result of the release and action of a variety of inflammatory factors. As an important part of its occurrence, adhesion molecules play a major role in mediating the adhesion of immune cells, as well as the migration to and the aggregation at the inflammatory sites (Zhong et al., 2018). CD34, also a member of the adhesion molecule family, has unique functions in addition to the interaction with other adhesion molecules during inflammatory diseases including IBD. For example, clinical studies revealed that CD34 and its family member vascular endothelial cadherin are used as markers for the immunofluorescence detection of inflammation (Xu et al., 2022). A further study showed that the expression of CD34 and CD146 in the intestinal mucosal endothelial cells of IBD patients is higher than that in the control group (Tsiolakidou et al., 2008). The expression of CD34 and platelet-derived growth factor receptor α (PDGFRα) in patients with Crohn’s disease is significantly increased in intestinal TCs that function to maintain the intestinal homeostasis (Milia et al., 2013). In addition, endothelial cells in the intestinal mucosa and gut-associated lymphoid tissue selectively expressing mucosal addressin cell adhesion molecule-1 also have CD34^+^ (Arihiro et al., 2002). These loads of evidence suggest the involvement of CD34 in the intestinal inflammation, as well as its role as a ligand for other adhesion factors. However, the specific mechanism regulating the connection with other adhesion molecules needs to be further investigated.

## 6 CD34^+^ cells and intestinal homeostasis

IBD is characterized by an impaired intestinal homeostasis, often leading to a decreased function of the intestinal immune barrier and an abnormal immune response (M'Koma, 2022; Ramos and Papadakis, 2019). The decrease in the above function and abnormal response is mostly caused by the decrease in the diversity of the intestinal microbiota and the increase pathogenic bacteria in the site of inflammation (Aldars-García et al., 2021), which is manifested by the direct or indirect contact and function of intestinal microbiota with immune cells. The balance of the intestinal homeostasis ensures the stability of the intestinal microbiota microenvironment. The surface of mast cells has a variety of intestinal microbiota receptors (De Zuani et al., 2018). Intestinal microbiota regulates their functions during the inflammatory response in IBD by contacting mast cells (Shimbori et al., 2022). Intestinal microbiota also regulates some functions of the eosinophils in the intestine, which are activated by IL-33 released by the intestinal microbiota, which in turn regulates the size of the intestinal villi and the integrity of the epithelial barrier (Ignacio et al., 2022). In addition, short-chain fatty acids secreted by the intestinal microbiota inhibit the production of inflammatory cytokines by neutrophils and improve mucosal inflammation in IBD mice (Li et al., 2021). Notably, the reduction of intestinal microbiota producing short-chain fatty acids is accompanied by a significant increase in the number of pro-inflammatory immune cells (Gonçalves et al., 2018). The stability of the intestinal microbiota microenvironment is essential for the maintenance of intestinal homeostasis and the development and outcome of intestinal inflammation.

Interstitial cells regulate intestinal function and are also closely related to the maintenance of the intestinal homeostasis. CD34 is used as a marker of ICC and TCs to evaluate their role in intestinal inflammation (Ricci et al., 2018; Wang et al., 2021); CD34^+^ TCs in the intestine during IBD significantly increase and are involved in eosinophil infiltration (Ricci et al., 2018); CD34^+^ ICC are decreased in ulcerative colitis, and ICC decrease is related to the increase of pathogenic bacteria in the small intestine during inflammation (Chen et al., 2017; Li et al., 2019). Correspondingly, the intestinal function is promoted by beneficial bacteria including *Clostridium* butyricum, which in turn promote the expression of cytokines in ICC (Sui et al., 2018). It is worth noting that a population of stromal cells, termed MRISC (also characterized by CD90^+^CD81^+^CD34^+^CD138^−^) is present in the healthy intestine and is a key component of the gut stem cell niche, facilitating the repair of intestinal damage (Wu et al., 2021). CD34^+^ stromal cells present in the intestine are plastic, since they behave as intestinal stem cells that differentiate into intestinal functional cells to maintain intestinal homeostasis (Melissari et al., 2021). Moreover, CD34^+^Gp38^+^ stromal cells in the intestinal submucosa constitute a microenvironment different from that of the intestinal microbiota. CD34^+^ cancer stem cells in the intestinal crypt release chemokines and recruit immune cells to the inflammatory site when the intestinal homeostasis is destroyed (Stzepourginski et al., 2017), promoting immune cells to restore the damaged intestinal homeostasis and improve the inflammatory symptoms. The regulation and the maintenance of the intestinal homeostasis provide new solutions for the treatment of IBD, and CD34^+^ cells can be used as a key target.

## 7 Discussion

Most of the current reports on CD34 focus on its role as a cell marker and disease predictor due to its advantages in molecular biology especially its stable molecular structure. Nevertheless, the role of CD34 in cell transport, colonization, signal transduction, intercellular adhesion, and inflammatory response has been gradually understood with the deepening of research and the progress of the detection methods. Despite that, the complete mechanism behind these functions still lacks systematic research. The molecular structure of CD34 suggests its potential role in the inflammatory response, since the sialylation site of the extracellular region of CD34 can work as a ligand for a variety of selectins, binding that initiates the inflammatory response (Krause et al., 1996). In addition, CrkL-SH3C recognizes the cytoplasmic localization of CD34 and induces cell aggregation. This is consistent with the role of CD34 in the promotion of the migration and aggregation of immune cells (Blanchet et al., 2007; Nielsen and McNagny, 2009; Aulakh, 2020). The above studies suggest that CD34 interacts with other molecules through its specific binding sites to activate cells or regulate their functions, suggesting the role of CD34 as a ligand participating in the inflammatory response.

IBD consists of a group of autoimmune diseases due to chronic intestinal inflammation. The immune response plays an important role in the course of IBD. The prevailing view is that IBD is caused by the dysbiosis of the intestinal microbiota in the gut, which leads to the impairment of the intestinal barrier function and induces abnormal immune responses in the intestinal site (Ramos and Papadakis, 2019; M'Koma, 2022). The dysbiosis caused by the increased proportion of intestinal pathogenic bacteria leads to the production of toxic factors and other metabolites by the pathogenic bacteria, which stimulate the intestine to release a large amount of inflammatory cytokines and chemokines (Stzepourginski et al., 2017) and induce abnormal immune responses in the body (Hills et al., 2019). The intestinal mucosal tissue is damaged under the action of a series of abnormal immune responses and develops inflammatory lesions. The abnormal expression or localization change of CD34 at the site of inflammation caught our attention. Studies have shown that immune cells such as neutrophils, eosinophils, and mast cells can migrate to the site of inflammation under the mediation of CD34 and participate in the inflammatory response process. However, the specific mechanism of CD34 in these processes is still lacking authoritative conclusions. In addition, the involvement of adhesion molecules such as selectins and integrins in the process of immune cell aggregation and adhesion cannot be separated from the participation of CD34. The current report on this process is that the extracellular domain of CD34 can be glycosylated and act as a ligand for three types of selectins, mediating the transmigration and aggregation of immune cells across vascular endothelial cells to the site of inflammation and participating in inflammatory responses. However, studies on the binding kinetics and mechanism of interaction between CD34 and selectins have not been reported. As for the functional influence of CD34 on integrins, CD34 can participate in the process of cell adhesion and cell surface structure formation mediated by individual integrins (Ohnishi et al., 2013; Hughes et al., 2020). The large amount of negative charge carried by CD34 enhances the adhesive ability of integrins and prevents non-specific adhesion (Lanza et al., 2001). However, many types of integrins exist, with different structures and functions, and the interaction between CD34 and the integrin family needs to be further studied. The mechanism regulating the interaction of CD34 with various adhesion molecules in IBD still needs further study, to further confirm the biological connection and significance. In recent years, the role of CD34^+^ stromal cells in the intestine has been continuously reported. Among them, the plastic cell subtypes contribute to the repair of damaged tissues in IBD inflammatory sites, but the specific mechanism remains unclear. Therefore, the investigation of the relationship between CD34 and IBD and the study of the synergistic mechanism between various immune cells/immune substances and CD34 at the inflammatory sites may help understand the pathogenesis of IBD and provide new solutions for its prevention and treatment. The resolution of these problems, both for the treatment of IBD and the control of morbidity, as well as the interpretation of CD34 function cannot be ignored.

## 8 Conclusion

**FIGURE 2:**
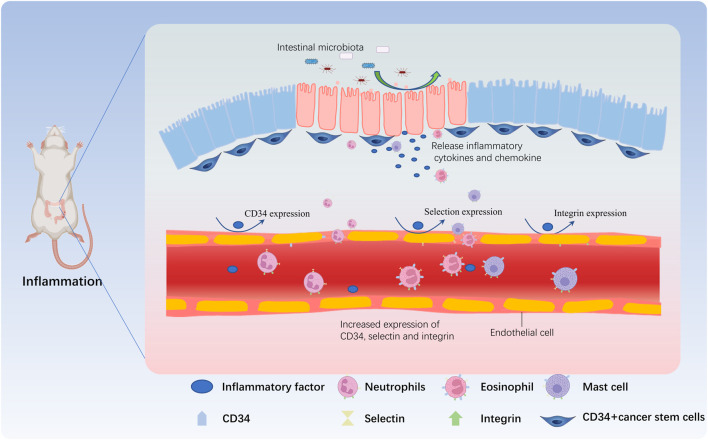
CD34 is involved in the immune regulation during IBD. When the proportion of harmful bacteria in the intestine increases, many toxic factors and other metabolites are released by harmful bacteria, damaging the intestinal mucosa. Intestinal goblet cells and CD34^+^ cancer stem cells release inflammatory factors and chemokines that act on vascular endothelial cells, and induce the expression of adhesion factors such as CD34, selectin, and integrin in vascular endothelial cells, increasing the permeability of the vascular wall. Neutrophils are the first reaching the inflammatory site due to the expression of CD34, and then mast cells and eosinophils further roll due to CD34 expressed by vascular endothelial cells and adhere to the inflammatory site by crossing the vascular wall, participating in the inflammatory response.

When the intestinal microbiota is dysregulated, a series of abnormal immune responses occur in the intestinal tissue, causing damage to the intestinal tissue, in turn induces intestinal inflammation. Under the combined stimulation of inflammatory cytokines and chemokines, the vascular permeability at damaged and inflamed site in the intestine increases, and the expression of CD34 and various adhesion molecules (e.g., selectins, integrins, etc.) on the surface of endothelial cells and various immune cells increases. Through the interaction between CD34 and selectins and/or integrins, immune cells roll, adhere and extravasate through sites of high permeability and accumulate at the damaged and inflamed sites, thereby triggering new or exacerbated inflammatory responses. In conclusion, the most common mechanism of migration of these immune cells involved in the inflammatory response (mainly neutrophils, eosinophils, and mast cells) to the site of inflammation depends on the interaction between CD34 and various adhesion molecules (Figure 2).
